# The land-grant mission in the 21st century: promises made and promises to be kept

**DOI:** 10.1093/af/vfaa016

**Published:** 2020-07-23

**Authors:** Stephen M Gavazzi

**Affiliations:** Department of Human Sciences, Ohio State University, Columbus, OH

**Keywords:** animal science, funding, land-grant universities, research, teaching

**Implications**

- The tripartite mission of land-grant universities (research, teaching, and extension) continues to produce astounding numbers of college graduates, inventions, and discoveries.
- Land-grant universities wishing to assert the benefits of a college degree must become more efficient, increase teaching excellence, engage with community stakeholders, conduct research that matters, describe how activities impact local needs, and refocus on affordability and accessibility.
- Additional public investment in animal science research, teaching, and extension activities are urgently needed.
- Animal scientists should consider how connected they are to the mission of land-grant universities that speaks directly to the 21st century needs of its partners and stakeholders.

## Introduction

**Figure 1.**
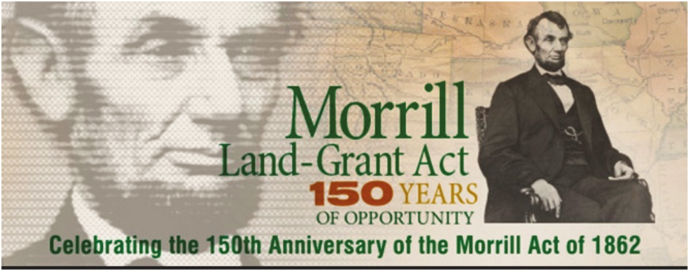
2012 marked the 150th anniversary of the Morrill Act, which established land-grant universities.

**Figure 2.**
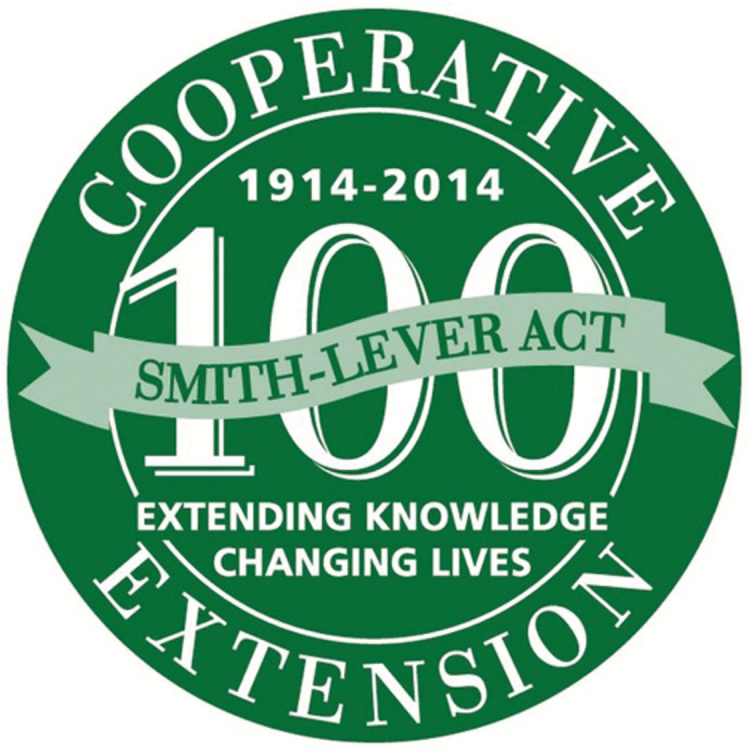
2014 marked the 100th anniversary of the Smith Lever Act, which created the Cooperative Extension System.

The year 2012 marked the sesquicentennial celebration of the Morrill Act of 1862 (Figure 1), the congressional action—signed by President Abraham Lincoln amidst the American Civil War—the gave rise to our nation’s land-grant universities. Coupled with two subsequent acts of the U.S. Congress—the Hatch Act of 1877 and the Smith-Lever Act of 1914 (Figure 2)—land-grant universities were assigned a tripartite mission by the federal government: to teach, to conduct research, and to provide service to communities. In the years that were to follow, this three-part mission produced astounding numbers of college graduates, countless inventions, and discoveries that have benefited society, and immeasurable societal benefits associated with work undertaken by Cooperative Extension Services personnel (Abramson et al., 2014).

**Figure 3.**
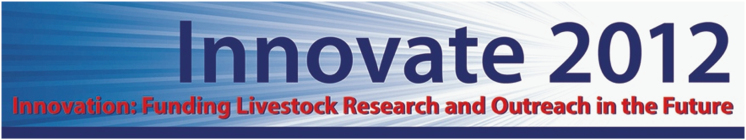
In 2012, the American Society of Animal Science launched the Innovate series of conferences with “Innovation: Funding Livestock Research and Outreach in the Future.”

During the same year that the nation was celebrating the sesquicentennial anniversary of the Morrill Land-Grant Act, the American Society of Animal Science (ASAS) implemented Innovate 2012 (Figure 3), an initiative designed to launch a national conversation about the future of teaching, research, and community engagement activities surrounding animal research. As reported by Benson et al. (2013), this effort sparked dialogue about “how public investments in agricultural research and development could be reinvigorated and concluded that land-grant universities have an opportunity to seek new and innovative partnerships with the private sector to support animal research.”

**Figure 4.**
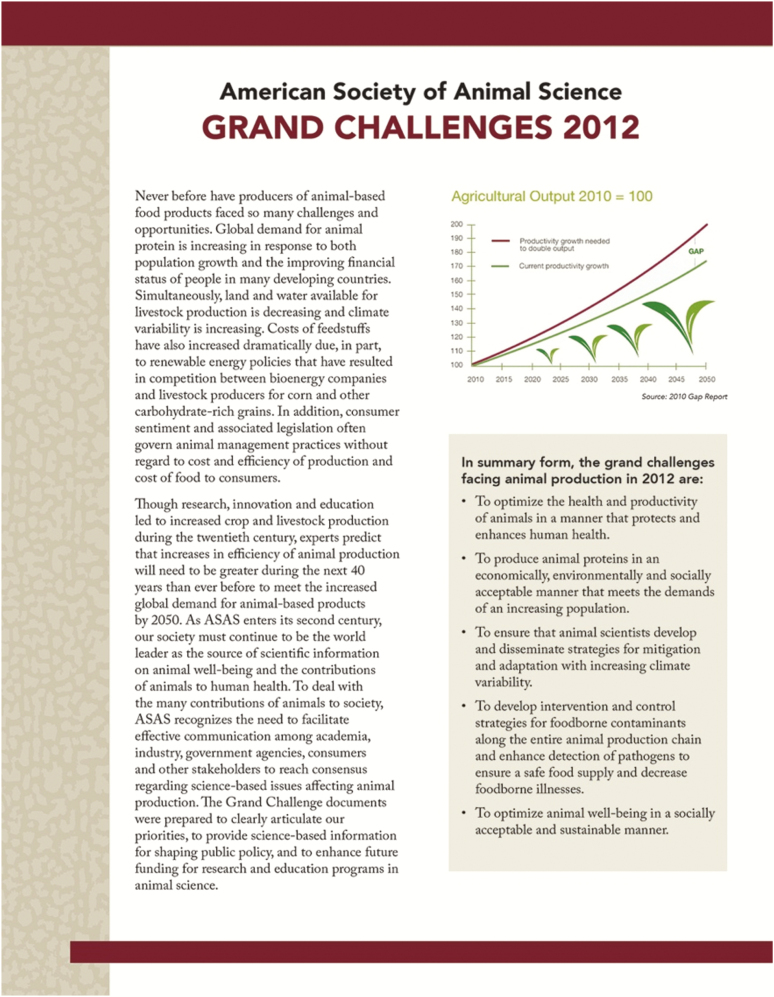
In 2012, the American Society of Animal Science developed Grand Challenges to clearly articulate priorities, to provide science-based information for shaping public policy, and to enhance future funding for research and education programs in animal science. More information is available at: https://www.asas.org/about/public-policy/asas-grand-challenges

One outcome of this national conversation was a set of “Grand Challenges” set forth by ASAS (Figure 4), an array of issues that included the need for an intensified focus on student enrollment in animal science programs at land-grant universities at a time when demand for animal scientists exceeded the available supply (ASAS, 2012). Since that time, several articles based on land-grant-oriented topics have appeared in *Animal Frontiers*, the official journal of ASAS. This literature has included an examination of data pertaining to students who have attended land-grant university animal science programs (Parrish et al., 2015), the role that land-grant universities have played in research breakthroughs and productivity growth in the pork industry (Tokach et al., 2016), and even the tracing of the African continent’s stagnant agricultural growth back to the failure of its universities to fully implement the American land-grant tripartite mission of teaching, research, and community engagement when it comes to the training of its meat producers (Dilger et al., 2016).

## The Land-Grant University Mission of the 21st Century

In 2018, Gavazzi and Gee published *Land-Grant Universities for the Future: Higher Education for the Public Good*, a book that reported on interviews conducted with 27 presidents and chancellors of America’s public land-grant institutions. These senior administrators were asked to comment on the strengths, weaknesses, opportunities, and threats facing land-grant universities in the 21st century. Analysis of the qualitative data gleaned from the interviews resulted in the development of seven themes that told a story about the dynamic tensions being faced by the leaders of these public universities, portrayed in dialectical fashion as follows:

1. Concerns about funding declines vs. the need to create efficiencies
2. Research prowess vs. teaching and service excellence
3. Knowledge for knowledge’s sake vs. a more applied focus
4. The focus on rankings vs. an emphasis on access and affordability
5. Meeting the needs of rural communities vs. the needs of a more urbanized America
6. Global reach vs. closer-to-home impact
7. The benefits of higher education vs. the devaluation of a college diploma

In addition to connecting these seven themes to the tripartite mission (teaching, research, and engagement) of the land-grant university, Gavazzi and Gee (2018) borrowed extensively from Robert Greenleaf’s (1970) discourse on servant leadership in order to introduce the concept of the “servant university.” Here, the authors strongly asserted the idea that public institutions of higher learning must place primary emphasis on the stewardship responsibilities they have been given by society to provide for the development and well-being of its communities. Here, the original agreement struck between the public and its colleges and universities—described invariably as a covenant—meant that critical decisions made at all levels of leadership should be filtered first through the lens of what provides maximum benefit for the citizens of each state and for American society at large. Better resemblance to the servant university profile, then, was asserted to be the defining path toward the creation and enactment of a 21st century land-grant mission.

## A Burning Platform for Land-Grant Universities

In 2011, then Nokia CEO Stephen Elop sent to his employees what has come to be known as his “burning platform” memo (Anthony, 2012). In this communication, Elop recounted the story of a North Sea oil worker who found himself quite literally on an offshore oil platform that was on fire. Faced with almost certain death had he stayed in place, he decided to jump from the platform and into the cold Atlantic waters. Elop wrote that “the man survived the fall and the waters. After he was rescued, he noted that a ‘burning platform’ caused a radical change in his behaviour.”

Here, we see a CEO signaling that his company was ablaze with challenges that required a course of action on the part of his employees that went far beyond what would be considered typical and usual. So, one must ask, are land-grant universities similarly standing on a burning platform at this moment in history? Gavazzi and Gee (2018) would have us believe so, pointing to a growing number of surveys that indicate our country’s citizens increasingly are skeptical about the importance of attending an institution of higher learning (Pew Research Center, 2017; Jaschik, 2019).

In response to this decided decline in public support, Gavazzi and Gee (2018) outlined a clear roadmap—discussed as a “formula for success”—that was designed to increase the public’s appreciation for the return on investment these higher learning institutions offered toward the public good. Nothing else would matter, these authors contended, unless land-grant universities reclaimed their mantle as the “people’s universities.” To do this, these public institutions of higher learning would have to “pick a side” in terms of the seven dialectical themes discussed above. As a result, universities wishing to assert the benefits of a college degree would have to become more efficient, cultivate increased teaching excellence, better engage with community stakeholders, conduct research that mattered, clarify how their university’s activities impacted the needs of local citizens (even amidst internationally based efforts), and refocus attention on being more affordable and accessible instead of worrying about national rankings.

## A Burning Platform for Animal Science?

This essay closes with a question: If animal scientists took a good look around right now, would they come to find themselves standing on a burning platform as well? Even a cursory examination of the goals and objectives related to the Innovate 2012 initiative would seem to provide at least some clues to that effect. Perhaps more substantially, the call made by Benson and colleagues (2013) regarding the need for reinvigoration of public investment in agricultural research and development speaks even more directly to an awareness that all is not right in animal science land at present. If this indeed is the case, then perhaps members of the field would do well to consider how connected they are to a land-grant mission that speaks directly to the 21st century needs of its partners and stakeholders.

## About the Author

**Stephen M. Gavazzi** is a professor in the human development and family science program in the College of Education and Human Ecology at The Ohio State University. He is the coauthor (with E. Gordon Gee) of *Land-Grant Universities for the Future: Higher Education for the Public Good*; *The Optimal Town-Gown Marriage,* and *Families with Adolescents: Bridging the Gaps between Theory, Research, and Practice*. Known primarily for his expertise regarding the impact that families have on adolescent development and well-being, Gavazzi more recently has developed a line of scholarship that examines campus–community relationship health and leadership issues in higher education.
